# Changes in the expression pattern of *OsWUS* negatively regulate plant stature and panicle development in rice

**DOI:** 10.1093/g3journal/jkad100

**Published:** 2023-05-04

**Authors:** Huiwu Tang, Jinlan Fan, Ru Wang, Jiahui Zhu, Xinzhu Xiang, Jingfang Dong, Lingyan Zhou, Limin Wang

**Affiliations:** Guangzhou Key Laboratory for Research and Development of Crop Germplasm Resources, College of Agriculture and Biology, Zhongkai University of Agriculture and Engineering, Guangzhou 510225, China; Guangzhou Key Laboratory for Research and Development of Crop Germplasm Resources, College of Agriculture and Biology, Zhongkai University of Agriculture and Engineering, Guangzhou 510225, China; Guangzhou Key Laboratory for Research and Development of Crop Germplasm Resources, College of Agriculture and Biology, Zhongkai University of Agriculture and Engineering, Guangzhou 510225, China; Guangzhou Key Laboratory for Research and Development of Crop Germplasm Resources, College of Agriculture and Biology, Zhongkai University of Agriculture and Engineering, Guangzhou 510225, China; Guangzhou Key Laboratory for Research and Development of Crop Germplasm Resources, College of Agriculture and Biology, Zhongkai University of Agriculture and Engineering, Guangzhou 510225, China; Rice Research Institute, Guangdong Academy of Agricultural Sciences/Guangdong Provincial Key Laboratory of New Technology in Rice Breeding, Guangzhou 510640, China; Guangzhou Key Laboratory for Research and Development of Crop Germplasm Resources, College of Agriculture and Biology, Zhongkai University of Agriculture and Engineering, Guangzhou 510225, China; Guangzhou Key Laboratory for Research and Development of Crop Germplasm Resources, College of Agriculture and Biology, Zhongkai University of Agriculture and Engineering, Guangzhou 510225, China

**Keywords:** rice, *OsWUS*, panicle development, dwarfism, gibberellic acid, Plant Genetics and Genomics

## Abstract

*WUSCHEL* (*WUS*) and *WUSCHEL*-*RELATED HOMEOBOX* (*WOX*) encode transcription factors and play important roles in regulating the formation and maintenance of shoot and floral meristems. *OsWUS* have distinct functions in meristem development with slightly tuned expression. However, the mechanisms regulating the specific expression of *OsWUS* need to be further explored. In this study, an abnormal expression mutant of *OsWUS*, called *Dwarf and aberrant panicle 1* (*Dap1*) was used. In order to identify the causal gene in *Dap1*, high-efficiency thermal asymmetric interlaced (hiTAIL)-PCR and co-segregation analysis were performed. We surveyed the growth and yield traits in *Dap1* and wild type. Changes in gene expression between *Dap1* and wild type were determined by RNA-seq. The *Dap1* mutant is due to the T-DNA inserted at 3,628-bp upstream of the translation start codon of *OsWUS*. Plant height, tiller numbers, panicle length, the number of grains per main panicle, and the number of secondary branches was significantly reduced in the *Dap1* mutant. The expression of *OsWUS* was markedly increased in *Dap1* mutant plants compared to the wild type, which might be due to a disruption in the genomic sequence integrity. Simultaneously, the expression levels of gibberellic acid-related genes and genes involved in panicle development were significantly changed in the *Dap1* mutant. Our results suggest that *OsWUS* is a precise regulatory element, its specific spatio-temporal expression pattern is critical for its function, and both loss-of-function and gain-of-function mutations lead to abnormal plant growth.

## Introduction

*WUSCHEL* (*WUS*), the prototypical member of the *WUSCHEL*-*RELATED HOMEOBOX* (WOX) gene family, encodes a homeodomain transcription factor that regulates the formation and maintenance of shoot- and floral meristems (FMs) (Laux *et al.* 1996; Mayer *et al.* 1998). The development of shoot apical meristems (SAMs) and FMs in plants is regulated by the WUS-CLV3 signaling pathway (Mjomba *et al.* 2016). *WUS* was originally identified in *Arabidopsis* (*AtWUS*) as a central regulator of cell fate in shoot and FMs. The WUS protein is produced in the organizing center (OC) domain of apical meristems (AM) and is transported to the stem cells of the central zone through plasmodesmata to promote the proliferation of stem cells (Yadav *et al.* 2011). *AtWUS* is a potential target involved in the *CLAVATA* (*CLV3*) pathway to regulate the fate of stem cells in meristems; the expression of *CLV3* negatively regulates *AtWUS* expression in the OC (Brand *et al.* 2000). The homeotic gene *AGAMOUS* (*AG*) is required for the formation and differentiation of FMs. *AtWUS* cooperates with the floral identity protein LEAFY to activate *AG* in the center of flowers, and the WUS and *AG* loop is important to control FM determinacy (Lenhard *et al.* 2001; Lohmann *et al.* 2001).

A single *WUS* ortholog was identified in rice based on phylogenetic analyses and was found to be expressed in young leaf primordia, preferentially in the lateral leaf margins (Nardmann and Werr 2006). *OsWUS* encodes a protein of 290 amino acids that contains a homeobox domain (HD) (Mjomba *et al.* 2016). *OsWUS*, also known as *TILLERS ABSENT1* (*TAB1*), is required for axillary meristem development. The *tab1* mutant produces no tillers during vegetative growth, but a single panicle with short branches is formed in *tab1* plants in the reproductive phase, and most of the spikelets show morphological defects (Tanaka *et al.* 2015). Another *OsWUS* mutant, *MONOCULM 3* (*moc3*) also produces no tillers because the formation of tiller buds is disrupted. Importantly, *moc3* is likely a female sterile mutant, because staining *moc3* pollen grains showed them to be viable (Lu *et al.* 2015). *STERILE AND REDUCED TILLERING 1* (*Srt1*), another mutant of *OsWUS*, produces a nearly full-length WUS peptide that is missing 7 amino acids in the HD. The *Srt1* mutant plants also showed a reduction in the number of tillers and complete female sterility. This report further demonstrated that the HD of OsWUS has a potential function in rice (Mjomba *et al.* 2016). The low-tillering mutant *decreased culm number 1* (*dc1*) was identified as a loss-of-function mutant of *OsWUS* by its increased apical dominance compared to wild type (WT). The auxin action-associated gene *ABERRANT SPIKELET AND PANICLE 1* (*ASP1*) and *OsWUS* are both involved in the outgrowth of the rice tiller bud (Xia *et al.* 2020).

Plant hormones (phytohormones) are low molecular weight signal molecules produced in plants that are active at extremely low concentrations. Phytohormones control essentially every aspect of growth and development by regulating gene expression, transcription, and cell division and differentiation. Cytokinins, a class of plant hormones that actively promote cell division in plant shoots and roots, are involved in the regulation of lateral bud growth and apical dominance. Cytokinins are required for the formation, maintenance, and growth of shoot meristems and act through a complex signaling network (Ferguson and Beveridge 2009). The *WUS* gene regulates tiller development in association with cytokinin signaling (Lu *et al.* 2015; Wang *et al.* 2017). In *moc3* plants, the expression levels of 2-component cytokinin response regulator genes (A-type and B-type), including *OsRR1*, *OsRR9*, *OsRR10*, and *ORR4* were found to be significantly up-regulated (Ito and Kurata 2006; Lu *et al.* 2015). Gibberellins (GA) play important roles in a variety of growth and developmental processes, including seed germination, stem elongation, flower development, apical dominance, plant stature, tillering, and root development (Ogas *et al.* 1999; Yamaguchi and Kamiya 2000; Oikawa *et al.* 2004; Lo *et al.* 2008; Huang *et al.* 2010). GA 20-oxidase (GA20ox) is a key enzyme that catalyzes the penultimate steps in GA biosynthesis (Lo *et al.* 2008). *OsGA20ox2* (*SD1*) is a member of the rice GA20ox gene family known as the “green revolution gene”, and loss-of-function mutations at this locus cause semi-dwarfism (Sasaki *et al.* 2002; Oikawa *et al.* 2004). A major catabolic pathway for GA is initiated by a 2-β hydroxylation reaction catalyzed by GA 2-oxidase (GA2ox). High expression levels of *OsGA2ox6* may cause decreased levels of bioactive GA, which could explain the dwarf phenotype in rice (Huang *et al.* 2010).

In this study, we isolated and characterized an abnormal expression mutant of *OsWUS* that we call *Dwarf and aberrant panicle 1* (*Dap1*). A T-DNA insertion 3,628-bp upstream of the translation start codon of *OsWUS* alters the expression pattern of *OsWUS* resulting in shorter plants with reduced panicle length and secondary branch number in the *Dap1* mutant. Transcriptomic analyses showed that the expression of GA-related genes and genes involved in panicle development and flowering regulation associated with *OsWUS* was significantly changed in the *Dap1* mutant. This uncoordinated gene expression may contribute to the phenotype of *Dap1* mutant plants.

## Methods

### Plant materials

The *Dap1* mutant was identified from a T-DNA insertion population created from the *japonica* rice variety ‘Zhonghua11’ (ZH11). All rice materials used in this study were grown in the field under normal growth conditions in Guangzhou City, China.

### T-DNA flanking sequence analysis

The T-DNA flanking sequences in the *Dap1* mutant were amplified by high-efficiency Thermal Asymmetric Interlaced (hiTAIL)-PCR (Liu and Chen 2007). The PCR products were purified and sequenced.

### Genotyping of mutant plants

To genotype the mutant plants, total DNA was isolated from T_2_-generation plants generated from a heterozygous T_1_ mutant plant. A T-DNA-specific primer (P1) and primers flanking the insertion site (P2 and P3) were used for PCR amplification (Supplementary Table 1). The primer pair P1/P2 directs amplification of a DNA fragment that includes part of 5′ end of the T-DNA and part of the upstream region of *OsWUS*, and only amplifies the transgene. Primer pair P2/P3 amplifies a 587-bp fragment of the upstream region of *OsWUS* from WT genomic DNA.

### Analysis of *cis*-regulatory elements in the upstream region of *OsWUS*

For analysis of the *cis*-regulatory elements in the sequence upstream of the T-DNA insertion site, we extracted a 500-bp fragment (from −4,128 to −3,629 bp) in the upstream region of *OsWUS* to detect possible *cis*-acting elements using New PLACE software (https://www.dna.affrc.go.jp/PLACE/?action = newplace) (Higo *et al.* 1999).

### RNA-seq analysis

RNA-seq analysis was conducted using total RNA extracted from leaves of *Dap1* mutant and WT plants at the booting stage with three biological replicates. Total RNA was extracted using TRIzol reagent (Invitrogen, Carlsbad, CA, USA) according to the manufacturer's instructions. RNA quality was assessed on an Agilent 2100 Bioanalyzer (Agilent Technologies, Palo Alto, CA, USA) and visually checked using RNase-free agarose gel electrophoresis. After total RNA extraction, the mRNA fraction was enriched with Oligo (dT) beads. The enriched mRNA was fragmented into short fragments using fragmentation buffer and then reverse transcribed into cDNA using the NEBNext Ultra RNA Library Prep Kit for Illumina (New England Biolabs, Ipswich, MA, USA). The purified double-stranded cDNA fragments were end repaired, a single base was added to the 3′ ends, and the fragments were ligated to Illumina sequencing adapters. The ligation reaction was purified with AMPure XP Beads (1.0×) and the DNA fragments were subjected to size selection by agarose gel electrophoresis and amplified using PCR. The resulting cDNA library was sequenced on an Illumina NovaSeq 6000 instrument by Gene Denovo Biotechnology Co. (Guangzhou, China).

In order to gain clean reads, the raw reads were filtered using Fastp by removing low-quality reads. We used the Bowtie 2 to remove the read of the rRNA on the comparison without mismatch and used the retained unmapped read for subsequent transcriptome analysis. The resulting high-quality clean reads were mapped to Nipponbare (http://plants.ensembl.org/Oryza_sativa/Info/Index) reference genome using HISAT 2. The resulting alignment was used Stringtie to reconstruct transcripts. The fragment counts of each gene were normalized by kb of transcript per million mapped reads to obtain the fragment per kilobase million (FPKM). Gene expression levels were estimated using FPKM values by the RSEM.

Gene annotation was performed using the NCBI nonredundant (Nr), Gene Ontology (GO) (http://www.geneontology.org/), and Kyoto Encyclopedia of Genes and Genomes pathway (KEGG) (https://www.kegg.jp/) databases. Differential expression analysis of mutant versus WT was performed using cuffdiff program, differentially expressed genes (DEGs) were identified using the software edgeR in pair-wise comparisons. DEGs among the samples were estimated by referring to the standard of false discovery rate (FDR) < 0.05 & | log2 (fold change) | > 1. Raw sequencing data have been uploaded in the NCBI Gene Expression Omnibus under the accession number PRJNA853805. Gene expression data are available at GEO with the accession number: GSE228728.

### RNA extraction and quantitative reverse-transcription PCR

Young panicles at booting stages from *Dap1* mutant and the wild type (ZH11) were sampled for qRT-PCR analysis. Young inflorescences (inflorescences length <3 cm) were collected and frozen at −80°C. For RNA extraction, each sample was grounded in liquid nitrogen. One microgram of RNA was subjected for first-strand cDNA synthesis with HiScript III RT SuperMix for qPCR (Vazyme, China). The cDNA products were used for qRT-PCR. *Actin1* (*Os03g0718100*) was used as an internal reference. Three independent biological replicates were performed. All the primers used for qRT-PCR analysis are listed in Supplementary Table 2. The qRT-PCRs were performed using the 7500 Real-Time PCR System (ABI, USA) and ChamQ Universal SYBR qPCR Master Mix (Vazyme, China). The qRT-PCR was conducted with an initial denaturation at 95°C for 3 min followed by 40 cycles of 95°C for 15 s, and 60°C for 30 s.

### Statistical analysis

Significance was determined by Student's *t*-test using Statistical Product and Service Solutions software (v19.0, IBM, USA). Two-sided tests were performed for homoscedastic matrices.

## Results

### Phenotype of the *Dap1* mutant

**Fig. 1.**
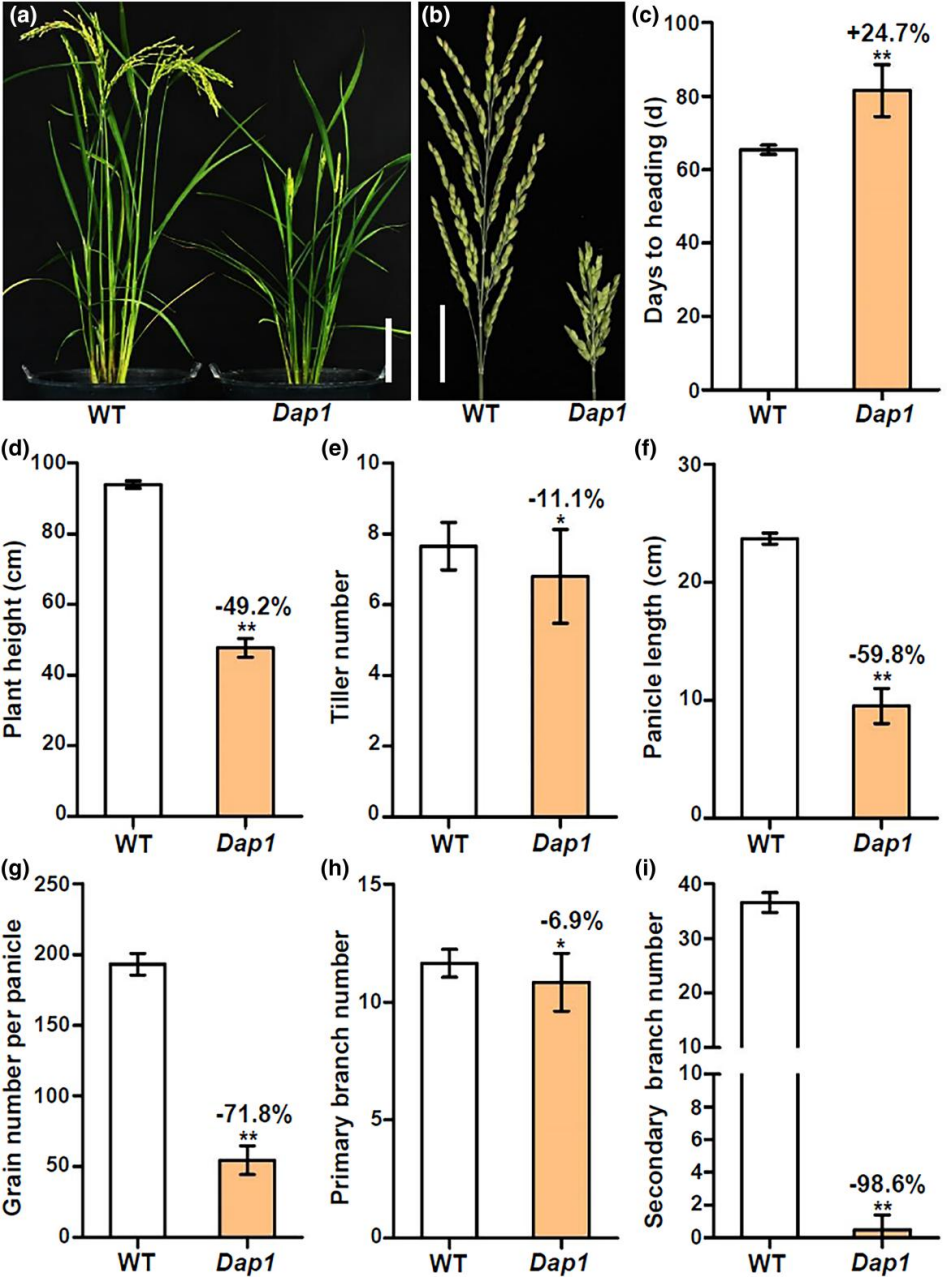
Phenotype of the *Dap1* mutant. a) Phenotypes of WT and *Dap1* mutant plants at the heading stage. Scale bar = 10 cm. b) The panicles of WT (ZH11) and *Dap1* plants. Scale bar = 3 cm. c–h) Quantification of plant height (c), tiller number (d), panicle length (e), grain number per panicle (f), primary branch number (g), and secondary branch number (h). Data are presented as the mean ± SD; *n* = 20; * *P* < 0.05; ** *P* < 0.01 as determined by Student's *t*-test.

The *Dap1* mutant was identified from a T-DNA insertion population created using the *japonica* rice variety ‘Zhonghua11’ (ZH11). In the T_2_ generation, the *Dap1* mutant and wild-type phenotypes segregated consistent with a 2:1 ratio (heterozygous *Dap1*:WT = 29:13, *χ*^2^ = 0.107, *P* > 0.05), and no homozygous *Dap1* seedling was identified in the T_2_ progeny (see below), suggesting that the mutant phenotype is controlled by a single dominant gene. The heterozygous *Dap1* mutant was selected for further phenotypic analysis. The growth and development of heterozygous *Dap1* mutant plants are slower than that of WT plants (Fig. 1a). Heading date of the *Dap1* mutants was delayed by more than 16 d compared with the WT plants (Fig. 1c). At the heading stage, the heterozygous *Dap1* mutant plants showed a 49.2% reduction in plant height and a severe enclosed-panicle phenotype compared to WT plants (Fig. 1a, b and d). Tiller numbers of *Dap1* mutant plants were decreased by 11.1% compared with WT plants (Fig. 1e). Furthermore, panicle length was significantly reduced by 59.8%, and the number of grains per main panicle in heterozygous *Dap1* plants was 71.8% lower than in WT plants (Fig. 1f and g). We found no significant differences in primary branch numbers between the mutant and WT (Fig. 1h); however, the number of secondary branches was significantly reduced by 98.6% (Fig. 1i) in the *Dap1* plants.

### A T-DNA insertion located 3,628 bp upstream of the *OsWUS* ATG is associated with the *Dap1* phenotype

**Fig. 2.**
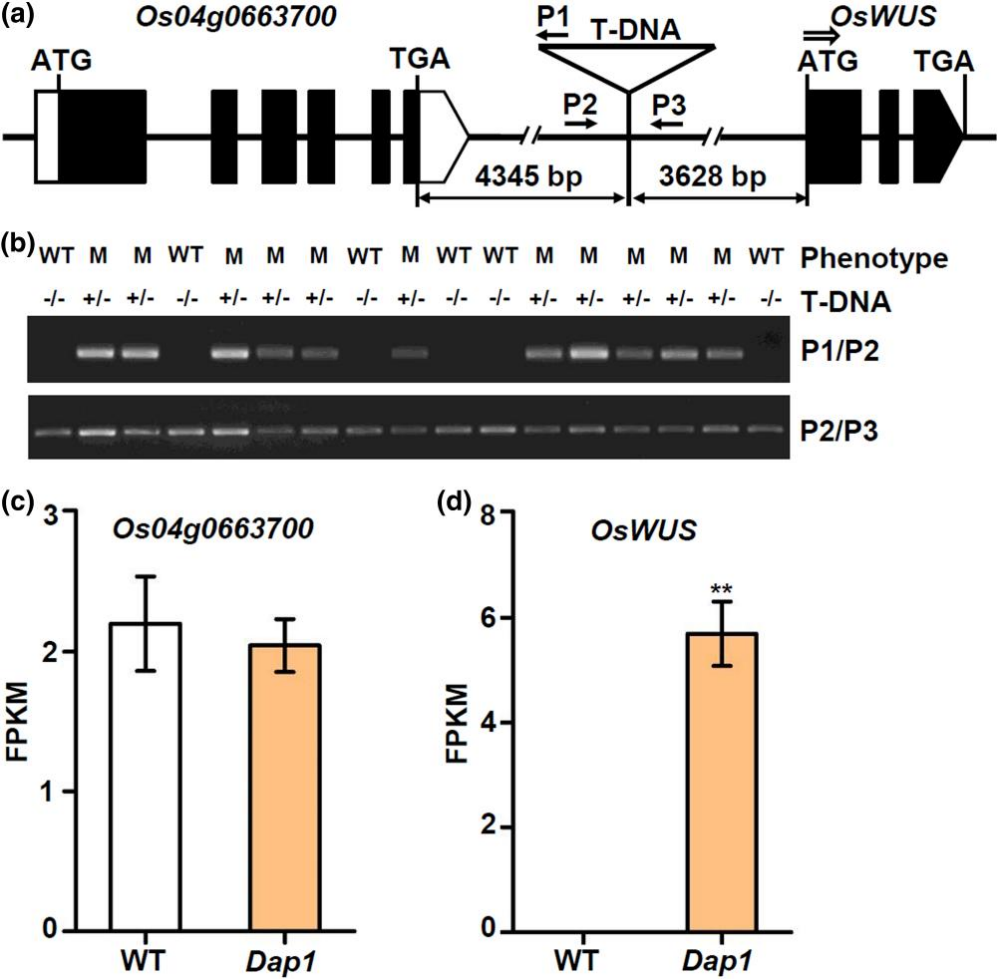
Molecular identification of the *Dap1* mutation. a) T-DNA insertion site in the genomic DNA and the 2 flanking genes, *OsWUS* (*Os04g0663600*) and *Os04g0663700*. Filled boxes represent coding exons, black lines represent the introns, and the white boxes indicate UTR regions. Rightwards double arrow indicate transcription start site of *OsWUS*. P1, P2, and P3 indicate the primers used for genotyping the hemizygous T-DNA insertions in the *Dap1* and WT plants descended from 1 hemizygous *Dap1* plant. b) Co-segregation of the T-DNA insertion in T_2_-generation progeny plants. −/− indicates no T-DNA insertion; +/− indicates plants hemizygous for the T-DNA insertion. c) Relative expression levels of *OsWUS* and *Os04g0663700* in leaves of WT and mutant plants at the booting stage determined by RNA-Seq analysis. Data are presented as the mean ± SD; *n* = 3; ** *P* < 0.01, according to Student's *t*-test. FPKM, fragments per kilobase of transcript per million mapped fragments.

To identify the causal gene in the *Dap1* mutant, we performed hiTAIL-PCR (Liu and Chen 2007) to recover genomic sequences flanking the T-DNA insertion. Sequencing analysis revealed that the T-DNA was positioned 3,628-bp upstream of the ATG of *OsWUS* (*Os04g0663600*), and 4,345-bp downstream of the TGA of a predicted gene called *Os04g0663700* (Fig. 2a). The T-DNA insertion also caused the deletion of a 77-bp fragment (from −3,705 bp to −3,629 bp) in the upstream region of *OsWUS*.

We next performed co-segregation analysis between the T-DNA insertion and the mutant phenotype in the T_2_ progeny using specific primers that anneal to the T-DNA (P1) and flanking genomic DNA sequences (P2 and P3). The 29 plants expressing the mutant phenotype were confirmed to be heterozygous (+//−) for the T-DNA insertion, while the other 13 plants with the normal phenotype were wild type (−//−) (heterozygous:WT = 2:1, *χ*^2^_2:1_ = 0.107, *P* > 0.05); however, no homozygous (+//+) plants were identified in the T_2_-generation plants (Fig. 2b). These results demonstrate that the *Dap1* mutant trait is due to a single T-DNA insertion and that it is inherited in a dominant fashion, and probably results from disruption or activation of the flanking gene(s).

To determine the causal gene for *Dap1*, we analyzed the expression of the 2 genes (*Os04g0663700* and *OsWUS*) that flank the T-DNA insertion in leaves from the mutant and WT plants at the booting stage. The results showed that there were no significant differences in the expression levels of *Os04g0663700* between WT and *Dap1* plants (Fig. 2c). However, the expression level of *OsWUS* in *Dap1* leaves was significantly higher than in WT leaves (Fig. 2d). The distinct expression patterns of *OsWUS* in the *Dap1* mutant compared to WT coincide with the mutant phenotype, which mainly affects plant stature and panicle development.

### Analysis of *cis-*regulatory elements in the upstream region of *OsWUS*

**Fig. 3.**
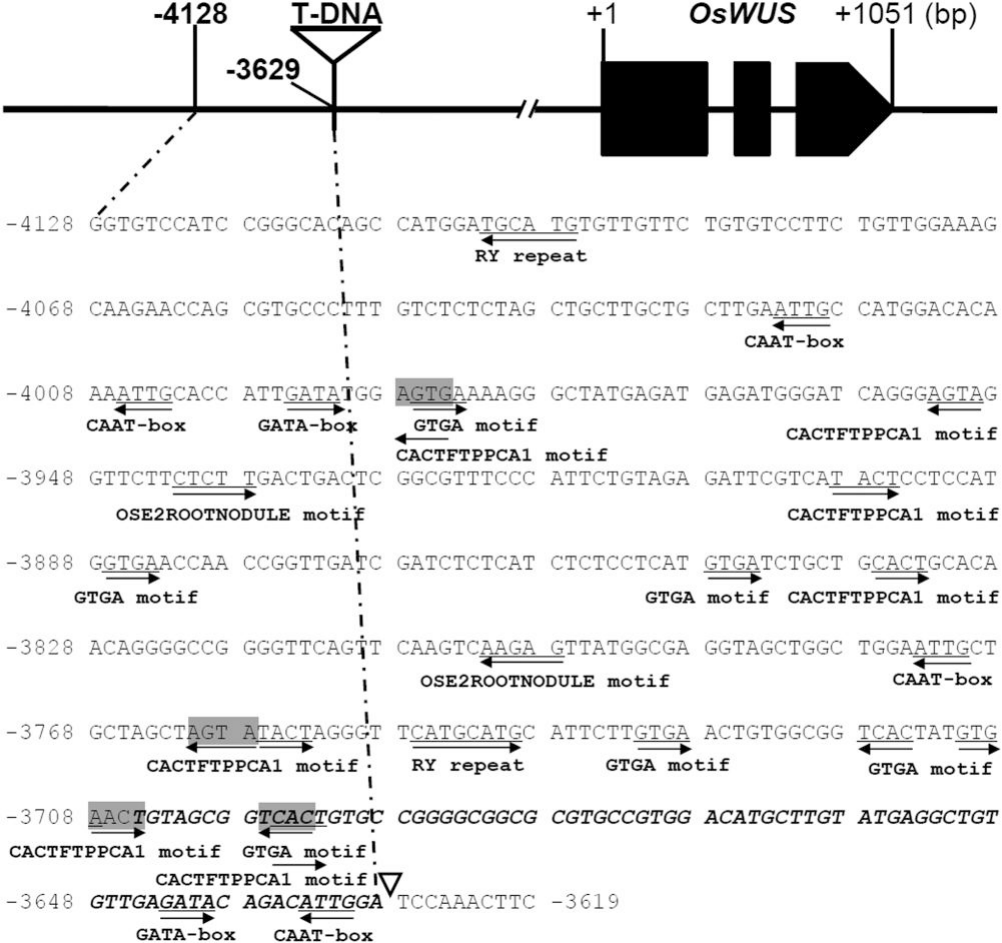
Putative *cis*-acting elements in the upstream of *OsWUS*. The “A” of the translation initiation code “ATG” of *OsWUS* was designated as “+1”. Putative *cis*-acting elements are shadowed and underlined. The arrowheads show their directions. Triangle indicates the T-DNA insertion site in *Dap1*. A fragment (−3,705 to −3,629 bp) was replaced by the inserted T-DNA in *Dap1* was shown in italics.

We reasoned that some *cis-*regulatory element(s) are probably present near the T-DNA insertion site between the *OsWUS* and *Os04g0663700* genes, and that they are essential for the specific pattern of *OsWUS* expression. Accordingly, we analyzed the upstream sequence of *OsWUS* using the online software New PLACE. The results showed that the 500-bp region upstream of the T-DNA insertion site contained a large number of *cis*-acting regulatory elements probably associated with precise spatial and temporal regulation of *OsWUS* expression. These included 2 GATA-box motifs, 7 GTGA motifs, 2 RY repeats, 4 CAAT-boxes, 8 CACTFTPPCA1 motifs, and 2 OSE2ROOTNODULE elements (Fig. 3). These results suggest that the altered expression pattern of *OsWUS* in the *Dap1* mutant might be due to a disruption in the genomic sequence integrity upstream of the gene.

### Transcriptomic changes between the *Dap1* mutant and wild type

**Fig. 4.**
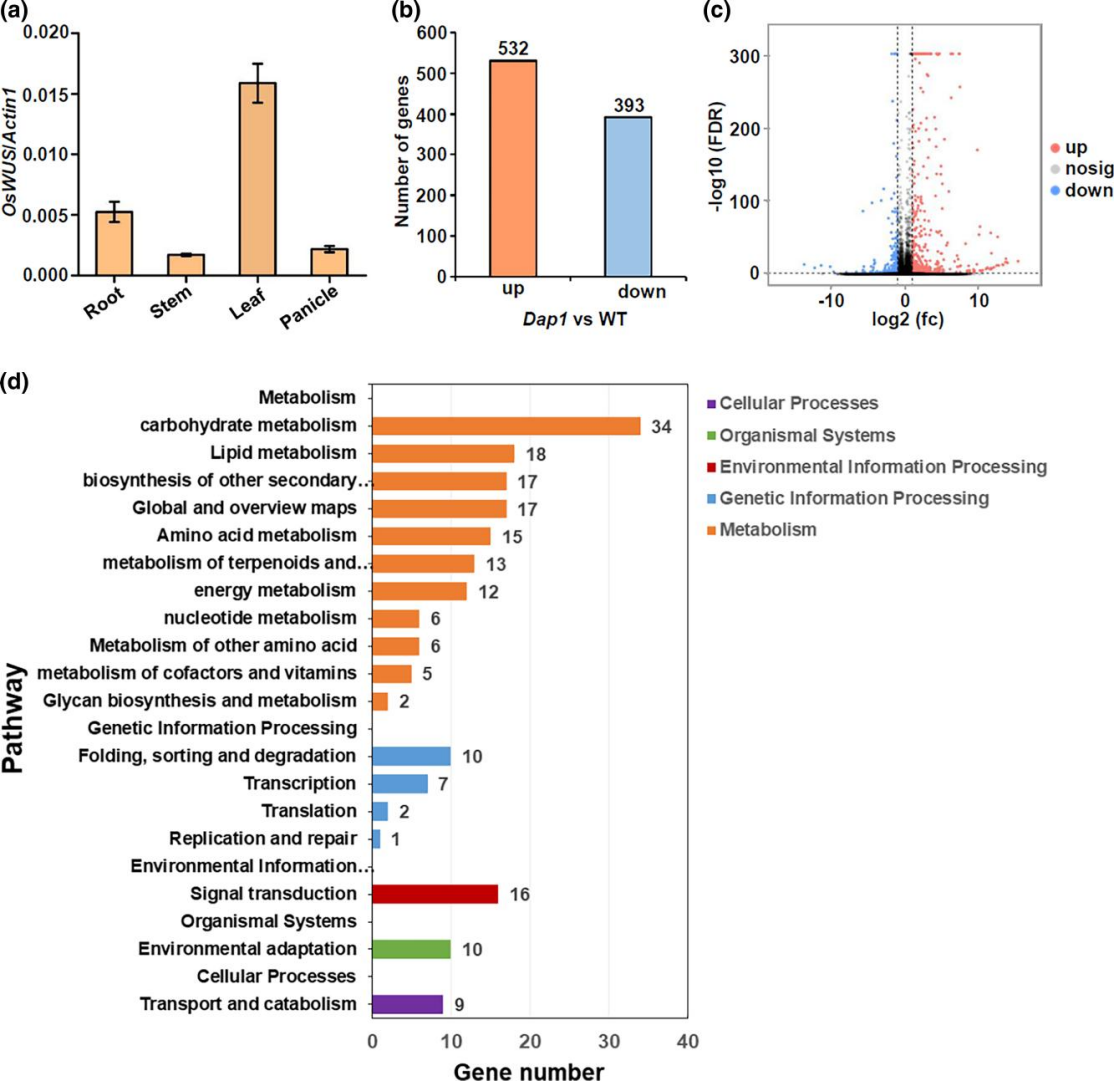
Global transcriptome changes in the *Dap1* mutant compared to WT (ZH11). a) Expression pattern analysis of *OsWUS* in wild type at booting stage by qRT-PCR. *Actin1* as an internal reference. b) The DEGs between *Dap1* and WT plants. The criteria for calling DEGs were a fold change ≥ 2.0 and FDR < 0.05. c) A volcano plot showing the DEGs in the *Dap1* mutant compared to WT. d) KEGG pathway enrichment analysis of the DEGs identified in the leaves of *Dap1* and WT plants at the booting stage (fold change ≥ 2.0, FDR < 0.05).

**Fig. 5.**
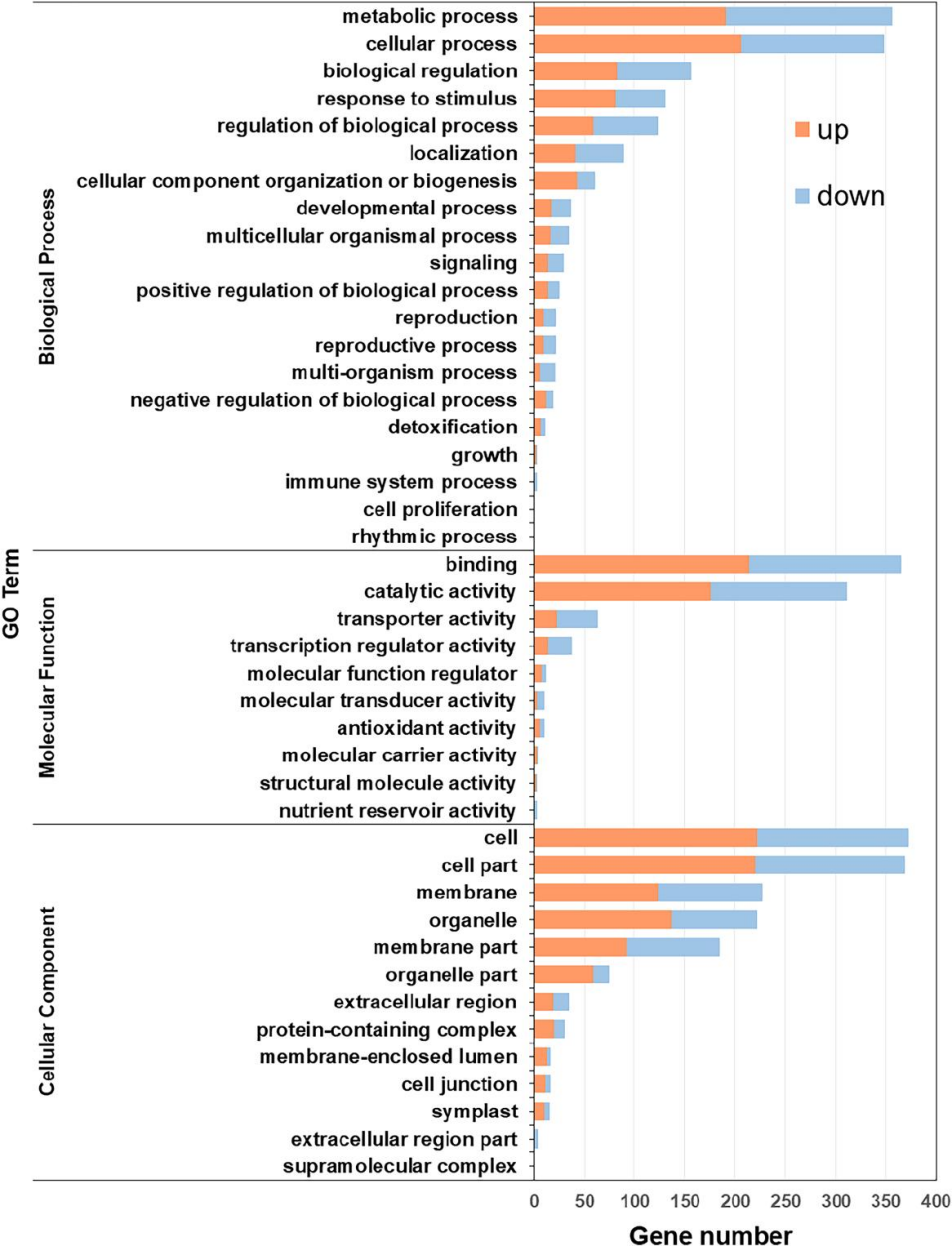
The top GO terms in the 3 main GO categories “BP”, “MF”, and “CC” for the DEGs in the leaves of *Dap1* and WT plants at the booting stage (fold change ≥ 2.0, FDR < 0.05).

**Fig. 6.**
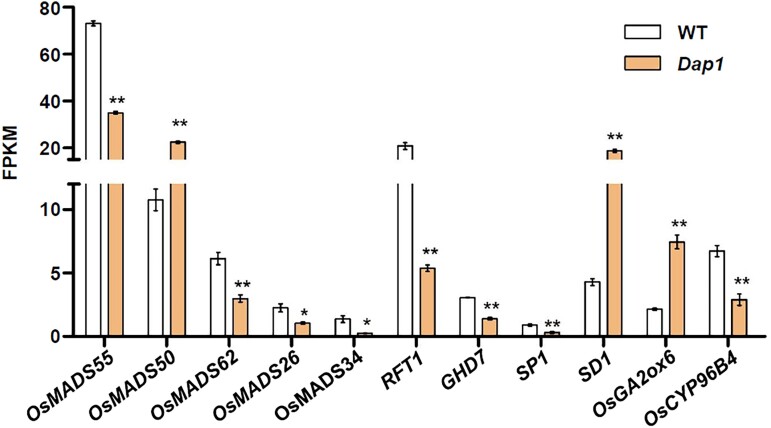
The expression level of genes involved in “floral development” in the leaves of *Dap1* and WT plants at the booting stage by RNA-Seq. Data are presented as the mean ± SD; *n* = 3; * *P* < 0.05; ** *P* < 0.01 as determined by Student's *t*-test.

To understand how *OsWUS* affects plant growth and panicle development, we examined its expression pattern using qRT-PCR. Expression pattern of *OsWUS* in roots, stems, leaves, and inflorescences of wild type were analyzed at the booting stage. The expression of *OsWUS* in leaves was relatively higher than that in other tissues (Fig. 4a). To elucidate the mechanism underlying the phenotype in the *Dap1* mutant, RNA-seq analysis was conducted to evaluate the changes in gene expression induced by the T-DNA insertion in the upstream region of *OsWUS*. Total RNA was extracted from leaves sampled from *Dap1* and WT (ZH11) plants at the booting stage. Three biological replicates were conducted for each sample. DEGs between the *Dap1* mutant and WT were estimated by referring to the standard of FDR < 0.05, |log2 fold change| > 1. A total of 925 genes in leaves were found to be differentially expressed in *Dap1*, with the expression of 532 genes significantly up-regulated and 393 genes down-regulated (Fig. 4b and c and Supplementary File 1). The DEGs identified between the *Dap1* mutant and WT plants were divided into 5 categories based on KEGG pathway enrichment; “metabolism”, “genetic information processing”, “environmental information processing”, “organismal systems”, and “cellular processes”. “Metabolism” represented the largest group, within which “carbohydrate metabolism” represented the largest subgroup (Fig. 4d). From GO enrichment analysis, the DEGs identified between the *Dap1* mutant and WT were classified into the 3 main ontologies “biological process” (BP), “molecular function” (MF), and “cellular component” (CC). The genes in the terms “metabolic process” and “cellular process” in BP, “binding” and “catalytic activity” in MF, and “cell”, “cell part”, and “membrane” in CC showed more changes in expression (Fig. 5). Short stature and abnormal panicle development are the 2 main characteristics observed in *Dap1* mutant plants. We selected genes related to plant stature and panicle development in the transcriptome and showed their relative expression in ZH11 and *Dap1* plants (Fig. 6 and Supplementary File 2). Panicle development in rice is regulated by multiple genes. The MADS-box gene family is found in all eukaryotic organisms and encodes transcription factors with a conserved DNA-binding domain, called the MADS box. This family includes more than 70 genes in rice (Arora *et al.* 2007; Lee *et al.* 2008). The expression level of *OsMADS50* in *Dap1* was higher than in the WT (ZH11), while the expression levels of *OsMADS55*, *OsMADS62*, *OsMADS26*, and *OsMADS34* were lower than in WT. The expression of other important flowering regulatory genes such as *RICE FLOWERING-LOCUS T 1* (*RFT1*), *HEADING DATE 7* (*GHD7*), and *SHORT PANICLE 1* (*SP1*) was also significantly lower than in WT (Fig. 6). Changes in the expression of these genes may contribute to the aberrant panicle phenotype in *Dap1* plants. Plant height is regulated by various factors, and gibberellic acid (GA) is one of the most important determinants of plant height (Sasaki *et al.* 2002). GA20-oxidase (GA20ox) is a key enzyme that catalyzes the late steps of gibberellin biosynthesis (Sasaki *et al.* 2002; Asano *et al.* 2007). The rice “Green Revolution” mutant gene *semi-dwarf1* (*sd1*), which has been widely used in breeding, encodes GA20-oxidase. The *sd1* mutant has a semi-dwarf phenotype due to the increased expression of *GA20ox2* (Sasaki *et al.* 2002). GA 2-oxidase plays a key role in the GA catabolic pathway through 2β-hydroxylation and regulates plant growth by inactivating endogenous bioactive gibberellins (Oikawa *et al.* 2004). The up-regulation of the *OsGA2ox6* gene may cause dwarfing by decreasing the levels of bioactive GA in the H032 mutant (Huang *et al.* 2010). Notably, the expression levels of *OsGA20ox2* (*SD1*) and *OsGA2ox6* were both found to be up-regulated in *Dap1* plants (Fig. 6). Plant cytochrome P450s are heme-binding enzymes with mono-oxygenase activities that are involved in a wide range of biosynthetic reactions. Plant cytochrome P450s constitute a large class of diverse enzymes that have played crucial roles in plant evolution. Due to their large number and substrate plasticity, the activities of plant P450s are central to many aspects of primary metabolism, including the biosynthesis of plant hormones and growth regulators such as gibberellins, jasmonic acid, auxin, and brassinosteroids (Nelson *et al.* 1996; Luo *et al.* 2006). Some of the genes encoding cytochrome P450 monooxygenases have been found to be involved in the regulation of plant height, such as *ELONGATED UPPERMOST INTERNODE1* (*Eui1*) and the CYP96 family (Luo *et al.* 2006; Ramamoorthy *et al.* 2011; Xie *et al.* 2018). The reduced expression level of *OsCYP96B4* in *Dap1* plants may also contribute to the dwarf phenotype (Fig. 6).

### The expression pattern of floral development-related genes in inflorescences changed in *Dap1* mutant

**Fig. 7.**
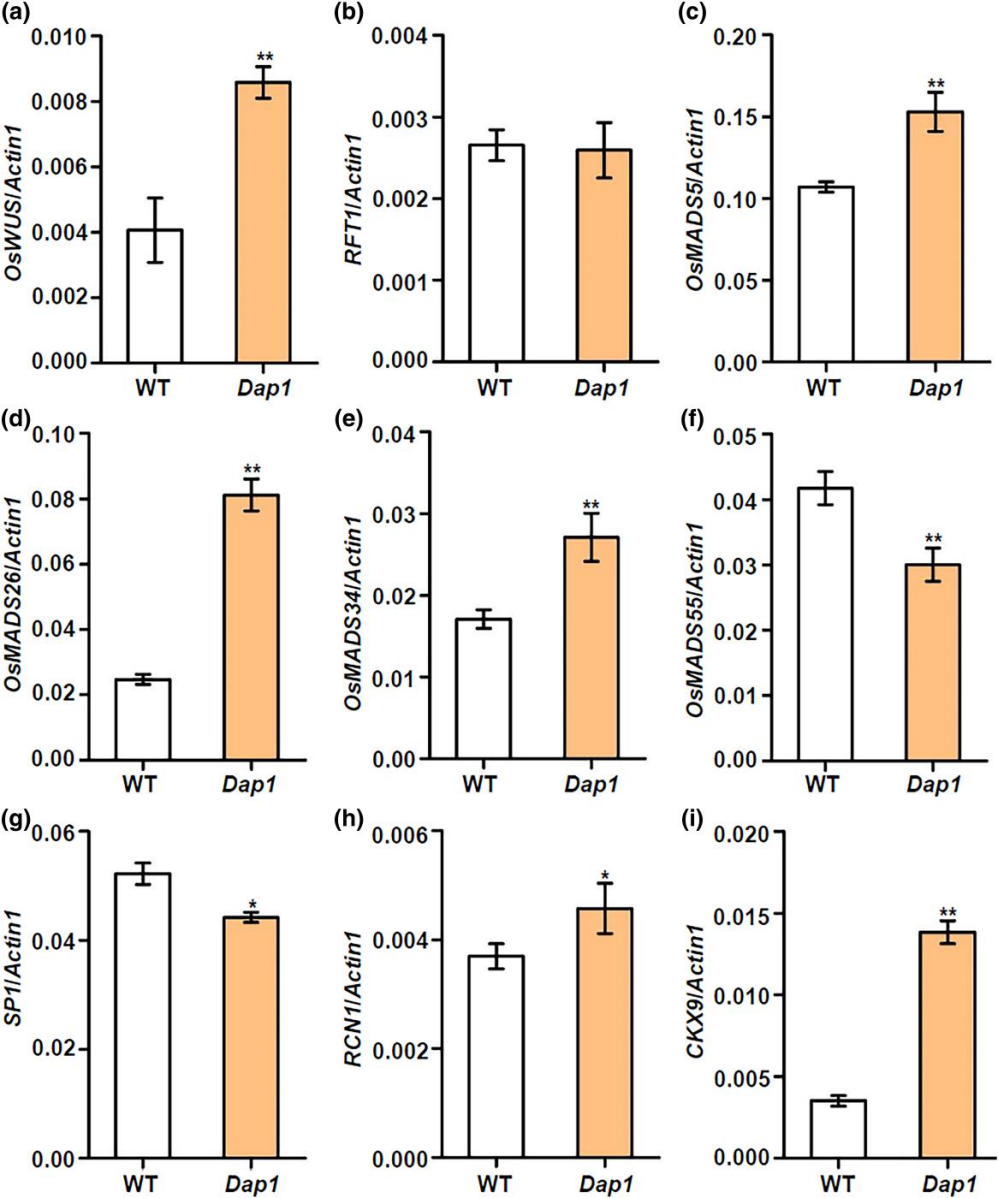
Expression levels of floral development related genes in inflorescences by qRT-PCR analyses at booting stage. *Actin1* as an internal reference. WT, wild type. Inflorescence length was <3 cm. a) *OsWUS*; b) *RFT1*; c) *OsMADS5*; d) *OsMADS26*; e) *OsMADS34*; f) *OsMADS55*; g) *SP1*; h) *RCN1*; i) *CKX9*. Data are presented as the mean ± SD; *n* = 3; * *P* < 0.05; ** *P* < 0.01, according to Student's *t*-test.

To determine the effect mechanism for *Dap1*, we further analyzed the expression alterations of *OsWUS* in the mutant and wild-type inflorescences (inflorescence length < 3 cm) at booting stages by qRT-PCR. The expression level of *OsWUS* in *Dap1* inflorescences was significantly higher than WT (Fig. 7a). We further examined the expression of genes related to panicle development in inflorescences. The expression level of *RFT1* in *Dap1* inflorescences was comparable to WT (Fig. 7b). *OsMADS5*, *OsMADS26*, and *OsMADS34* expressed in *Dap1* inflorescences were elevated compared to WT, while the expression levels of *OsMADS55* were lower than WT (Fig. 7c–f). Simultaneously, the expression of *SP1* in *Dap1* inflorescences was significantly lower than WT (Fig. 7g). *TERMINAL FLOWER 1* (*TFL1*)/*CENTRORADIALIS* (*CEN*)-like genes play important roles in determining plant architecture, mainly by controlling the timing of phase transition (Nakagawa *et al.* 2002). Expression level of *RCN1* (rice *TFL1*/*CEN* homologs) in *Dap1* inflorescences was increased compare to WT (Fig. 7h). Previous studies have shown that the *WUS* gene regulates growth and development in association with cytokinin signaling (Lu *et al.* 2015; Wang *et al.* 2017). *CYTOKININ OXIDASE/DEHYDROGENASE 9* (*OsCKX9*) encodes a cytokinin oxidase to catalyze the degradation of cytokinin, functions as a primary strigolactone-responsive gene to regulate rice tillering, plant height, and panicle size (Duan *et al.* 2019). We found that the expression level of *OsCKX9* in *Dap1* inflorescences was significantly increased (Fig. 7i).

## Discussion

*OsWUS*, a rice ortholog of *Arabidopsis WUSCHEL* (*WUS*), plays a crucial role in tiller bud formation and development in rice (Lu *et al.* 2015; Tanaka *et al.* 2015; Mjomba *et al.* 2016). There are currently 4 *OsWUS* loss-of-function mutants reported in rice, they are *monoculm 3 (moc3), tillers absent 1 (tab1), sterile and reduced tillering 1 (Srt1), and decreased culm number1 (dc1)*. *moc3* and *tab1* are 2 null allelic mutants of *OsWUS*, which result in partial N-terminal peptides of OsWUS. The Srt1 mutant protein has a deletion of 7 amino acids in the conserved HD of OsWUS, while the *dc1* mutation results in a truncated protein that lacks the WUS-box and EAR motif at the C-terminus. These 4 mutants show similar plant phenotypes, such as no or fewer tillers, malformed spikelets, and female sterility. In addition, *Srt1* plants show an opposite effect on panicle development compared to the other 3 mutants, and the panicles of *Srt1* are larger than those of WT (Lu *et al.* 2015; Tanaka *et al.* 2015; Mjomba *et al.* 2016; Xia *et al.* 2020). These observations confirm that the loss of *OsWUS* function has a serious impact on AM formation and its subsequent outgrowth. *TAB1*/*OsWUS* plays an important role in the formation of AMs by regulating *OSH1* expression (Tanaka *et al.* 2015). Tanaka and Hirano (2020) further showed that *TAB1* is required for maintaining stem cells during axillary meristem development, and *FON2* (*FLORAL ORGAN NUMBER2*) negatively regulates stem cell fate by restricting *TAB1*. MOC3/OsWUS is a vital regulator of tiller formation in rice and is able to directly bind the promoter of *FLORAL ORGAN NUMBER1* (*FON1*) and subsequently activate the expression of *FON1*, the homolog of *CLAVATA1* (Shao *et al.* 2019). In *dc1* plants, OsWUS and the auxin action-associated gene *ASP1* are both involved in the outgrowth of tiller buds in rice, and a transcription factor that putatively binds to *ORYZA SATIVA HOMEOBOX 1* (*OSH1*) also participates in the regulation (Xia *et al.* 2020).

In this study, we have identified a T-DNA insertion mutant of *OsWUS* that differs from the previously reported mutants. In the *Dap1* mutant, the T-DNA insertion is flanked by 2 genes, *OsWUS* and *Os04g0663700*. There was no significant difference in the expression level of *Os04g0663700* between *Dap1* and the WT (Fig. 2c). However, the expression level of *OsWUS* in *Dap1* leaves was significantly higher than in leaves of WT plants (Fig. 2d). This indicated that the T-DNA insertion mainly affects the expression of *OsWUS*, resulting in a different plant phenotype compared to the WT (Fig. 1a). Alteration or disruption of regulatory sequences can change gene expression patterns and produce developmental phenotypes. For example, the multiple copies of the AGATAT element present in the proximal promoter region of the maize *ZmWUS1-B* gene cause its overexpression, and lead to major rearrangements of inflorescence meristems and mis-regulation of key stem cell regulators (Chen *et al.* 2021). Sequence analysis showed that the 500-bp region upstream of the T-DNA insertion site harbors 2 RY elements (Fig. 3). The RY elements are binding sites for B3 domain-containing proteins involved in transactivation. The rice B3 domain-containing transcription factor OsGD1, a homolog of *Arabidopsis* VAL proteins, suppresses *OsLFL1* expression by binding to the RY element in the *OsLFL1* promoter and is associated with seedling development and GA homeostasis in rice (Guo *et al.* 2013). Moreover, the B3 transcriptional repressors OsGD1 and OsVAL2 bind to the RY-containing *cis*-silencing element (*SE1*) in the first intron of the rice GA-deactivating enzyme gene *Eui1* and recruit a *trans* epigenome reader that represses *Eui1* expression and modulates GA homeostasis. Deletion of *SE1* elevates the expression of *Eui1* in the *dEui1* mutant, thus leading to GA deficiency and dwarfism (Xie *et al.* 2018). The *Dap1* mutant exhibited a dwarf phenotype and abnormal panicle development, which is similar to *gd1* and *dEui1*. In summary, we conclude that the altered expression pattern of *OsWUS* in *Dap1* is probably due to a disruption in the genomic sequence integrity upstream of the gene.

Analysis of RNA-seq data identified a total of 925 genes that were differentially expressed in leaves between the *Dap1* mutant and WT (Fig. 4a and b). In the KEGG pathway enrichment analysis, “metabolism” represented the largest group, in which “carbohydrate metabolism” represented the largest subgroup (Fig. 4c). From the GO enrichment analysis, the largest number of DEGs were in the “metabolic process” term in the “BP” category, “binding” in “MF”, and “cell” in “CC” (Fig. 5). The panicle architecture of *Dap1* plants was characterized by short, malformed panicle with little seed set (Fig. 1b). Several genes related to floral development were abnormally expressed in *Dap1* plants. *OsMADS50* plays an important role in regulating flowering time in rice. Inhibiting the expression causes late flowering, while ectopic expression of *OsMADS50* causes early flowering (Lee *et al.* 2004). *OsMADS34* [also called *PANICLE PHYTOMER2* (*PAP2*)] is induced in the SAM during the transition from vegetative to reproductive development and is also induced when glumes are initiated during spikelet development (Kobayashi *et al.* 2012). The decrease in the expression of *OsMADS34* in *Dap1* may affect the initial development of the panicle. At the same time, *OsMADS34* acts immediately downstream of *RFT1* (Pasriga *et al.* 2019). *RFT1* is a major florigen, and its function is to induce reproductive development of the SAM. The expression of both *RFT1* and *OsMADS34* was also reduced in the *Dap1* mutant (Fig. 6). *GHD7* has a key role in photoperiodic flowering by regulating the putative *Ehd1*-*Hd3a* pathway and has positive regulatory effects on an array of traits in rice, including the number of grains per panicle, plant height, and heading date under long-day conditions (Xue *et al.* 2008). Although rice is a short-day plant, its domestication led to the *Ghd7-Ehd1-Hd3a/RFT1* pathway for adaptation to long-day conditions (Peng *et al.* 2021). We showed that *GHD7* and *RFT1* are both down-regulated in *Dap1* leaves (Fig. 6), and these may contribute to the delayed flowering phenotype in *Dap1* mutants (Fig. 1a and c).

Expression pattern of genes related to floral development in inflorescences were further determined. The expression levels of the MADS-box family genes in inflorescences were significantly changed between *Dap1* and WT (Fig. 7). *OsMADS5* and *OsMADS34* expressed in *Dap1* inflorescences were higher than that in WT (Fig. 7c and e). *OsMADS5* and *OsMADS34* play similar functions in limiting branching and promoting the transition to spikelet meristem identity. The number of primary branches per panicle in *OsMADS5* and *OsMADS34* overexpression lines was comparable to WT plants; however, the number of secondary branches and spikelets decreased significantly (Zhu *et al.* 2022). Here, we observed similar phenotypes in *Dap1* mutant (Fig. 1h and i). The expression of *OsMADS34* in leaves was reduced (Fig. 6), but this expression in inflorescences was significantly increased in the *Dap1* mutant (Fig. 7e). It seems that the mutation delays the initial vegetative to inflorescence meristem transition (Fig. 1a and c), but later accelerate the transition from branching to spikelet meristems, resulting in less secondary branching (Fig. 1b and i). The architecture of the rice inflorescence, which is determined mainly by the number and length of primary and secondary inflorescence branches. *SP1* encodes a PTR family transporter and determines rice panicle size (Li *et al.* 2009). *OsCKX9* plays a critical role in regulating rice shoot architecture, and *OsCKX9*-overexpressing transgenic plants showed significant decreases in plant height and panicle size (Duan *et al.* 2019). In *35S*::*RCN1* transgenic rice plants, the delay of transition to the reproductive phase was observed and the transgenic plants exhibited a more branched, denser panicle morphology (Nakagawa *et al.* 2002). We found that expression level of *OsCKX9* and *RCN1* both increased in *Dap1* plants (Fig. 7h and i). The T-DNA insertion in the *Dap1* mutant causes the overexpression of *OsWUS* in leaves and panicles, and coordinated regulation of these genes contributes to the abnormal panicle development in *Dap1* plants.

In previous reports, the expression of *OsWUS* in association with cytokinin signaling was shown to regulate tiller development in rice (Lu *et al.* 2015; Wang *et al.* 2017). In our study, we first found that the overexpression of *OsWUS* can regulate plant stature in association with gibberellic acid biosynthesis. GA 20-oxidase (GA20ox) is a 2-oxoglutarate-dependent dioxygenase and is a key enzyme in the biosynthesis of gibberellins (Oikawa *et al.* 2004). The mutant gene *semi-dwarf1* (*sd1*), which is known as the “Green Revolution” gene, encodes GA 20-oxidase and is one of the most important genes used in rice breeding. The *sd1* mutation affects the late steps in gibberellin metabolism, leading to an accumulation of the initial substrate of GA20ox, low gibberellin levels, and a semi-dwarf phenotype (Sasaki *et al.* 2002; Spielmeyer *et al.* 2002). Gibberellin (GA) 2-oxidase plays a key role in the GA catabolic pathway through 2β-hydroxylation (Huang *et al.* 2010). GA2ox6 is localized to the nucleus and cytoplasm, and the mRNA can be detected in young seedling leaves and transiently at high levels during the active tillering stage, which mainly affects plant stature (Lo *et al.* 2008; Huang *et al.* 2010). In our study, the expression levels of *OsGA20ox2* (*SD1*) and *OsGA2ox6* in the *Dap1* mutant were both elevated compared to WT (Fig. 6). In addition, expression of *OsCYP96B4* was lower in *Dap1* plants than in WT (Fig. 6). A point mutation in the SRS2 domain of *CYP96B4* resulted in a dwarf phenotype in the *sd37* mutant (Zhang *et al.* 2014). Another *CYP96B4* mutant, *dss1*, also shows a dwarf phenotype, and this phenotype is regulated by finetuning the GA-to-ABA balance (Tamiru *et al.* 2015). In addition, the bioactive forms of GA (GA_1_ and GA_4_) could not be detected in *dss1* mutant, but the levels of the GA precursors (GA_19_ and GA_53_) were significantly reduced (Tamiru *et al.* 2015). These results show that the deficiency of *OsCYP96B4* leads to the decrease of accumulation of bioactive GAs. Accumulation of bioactive GAs triggers GA signaling pathways and inhibits GA biosynthesis, whereas deficiency of bioactive GA upregulates GA biosynthetic enzymes and inhibits GA catabolic enzymes via feedback regulation (Fleet and Sun 2005). Therefore, the feedback regulation of deficiency of bioactive GA may contribute to higher expression of *OsGA20ox2* (*SD1*) in *Dap1* mutants.

ConclusionsIn this study, we identified a T-DNA insertion mutant that causes the overexpression of *OsWUS*, which resulted in some phenotypes similar to that of a loss-of-function *OsWUS* mutant. Furthermore, our results indicate that *OsWUS* is an essential panicle development regulator in rice, and the abnormal expression of *OsWUS* mutation is associated with the gibberellin signaling pathway to regulate plant stature.

## Supplementary Material

jkad100_Supplementary_Data

## Data Availability

The data that support the findings of this study are available in the Supplementary material of this article. Supplemental data are available at G3 online. Supplementary Files 1 and 2 contain DEGs between the *Dap1* mutants and WT plants by RNA-seq analysis. Raw sequencing data have been uploaded in the NCBI Gene Expression Omnibus under the accession number PRJNA853805. Gene expression data are available at GEO with the accession number: GSE228728. Supplemental material available at G3 online.
