# Supplementary material for: Changes in the expression pattern of OsWUS negatively regulate plant stature and panicle development in rice
Source: G3 (Bethesda). 2023 May 4;13(7):jkad100. doi: 10.1093/g3journal/jkad100 (PMC10320761; doi:10.1093/g3journal/jkad100)
Supplement: jkad100_Supplementary_Data [file jkad100_supplementary_data.zip › Table_S1_G3-2022-404011.docx]

**Table S1. Primers used** **for hiTAIL-PCR**

| **Primers** | **Sequence (5’-3’)** | **Usage and notes** |
| --- | --- | --- |
| P1 | CTGTTGCCGGTCTTGCGATGA | Co-segregation analysis  T-DNA specific primer |
| P2 | GCTATGAGATGAGATGGGATCA | Co-segregation analysis  Primer flanking T-DNA insertion site |
| P3 | CGTGTGGCAAGTGATCAATTGA | Co-segregation analysis  Primer flanking T-DNA insertion site |
