# Supplementary material for: Changes in the expression pattern of OsWUS negatively regulate plant stature and panicle development in rice
Source: G3 (Bethesda). 2023 May 4;13(7):jkad100. doi: 10.1093/g3journal/jkad100 (PMC10320761; doi:10.1093/g3journal/jkad100)
Supplement: jkad100_Supplementary_Data [file jkad100_supplementary_data.zip › Table_S2_G3-2022-404011.docx]

Table S2. Primers used in this study.

| Name | Forward Primer（5′-3′） | Reverse Primer（5′-3′） |
| --- | --- | --- |
| *OsWUS* | tgcagacggatcaggccaacg | gccgctcacgcccatgtagt |
| *RFT1* | CCGTCTACTTCAACTGCCAG | GTCTCAGCTTAGCTATAGCT |
| *OsMADS5* | GCTTCATATATCTTGCCAAG | TTGGTTGAGGTGATCCATGT |
| *OsMADS26* | GCTCGGAGATGGGCTATTCCTTC | GACACTTCCTGCGGGAACTTGTC |
| *OsMADS34* | TTGATGAACTCTGCGACCTAAA | TGCTGCAGTTTCCGTTCC |
| *OsMADS55* | TGGAAGAGCTGCAGCAGATG | TCATCACAGATTCAGATGATTG |
| *SP1* | TTCAACGCGGCCTACTTCAG | CGATGATGATGGGCACACTT |
| *CKX9* | CTATCCTCAGCACTTGGCCC | AAATGGGACTGCCACTCCTG |
| *RCN1* | GCTCACCGAAGTGCATTAGC | TCACTTGGTCCTGGCACATC |
| *Actin1* | gcatctctcagcacattcca | ACCACAGGTAGCAATAGGTA |
